# Supplementary material for: Unified bursting strategies in ectopic and endogenous even-skipped expression patterns
Source: bioRxiv. 2024 Jun 25:2023.02.09.527927. Originally published 2023 Feb 10. Preprint. [Version 2] doi: 10.1101/2023.02.09.527927 (PMC9934701; doi:10.1101/2023.02.09.527927)
Supplement: Supplement 1 [file NIHPP2023.02.09.527927v2-supplement-1.pdf]

bioRxiv preprint doi: https://doi.org/10.1101/2023.02.09.527927; this version posted June 25, 2024. The copyright holder for this preprint (which was not certified by peer review) is the author/funder, who has granted bioRxiv a license to display the preprint in perpetuity. It is made available under aCC-BY 4.0 International license.

Supplemental Information

Supplemental Figure 1

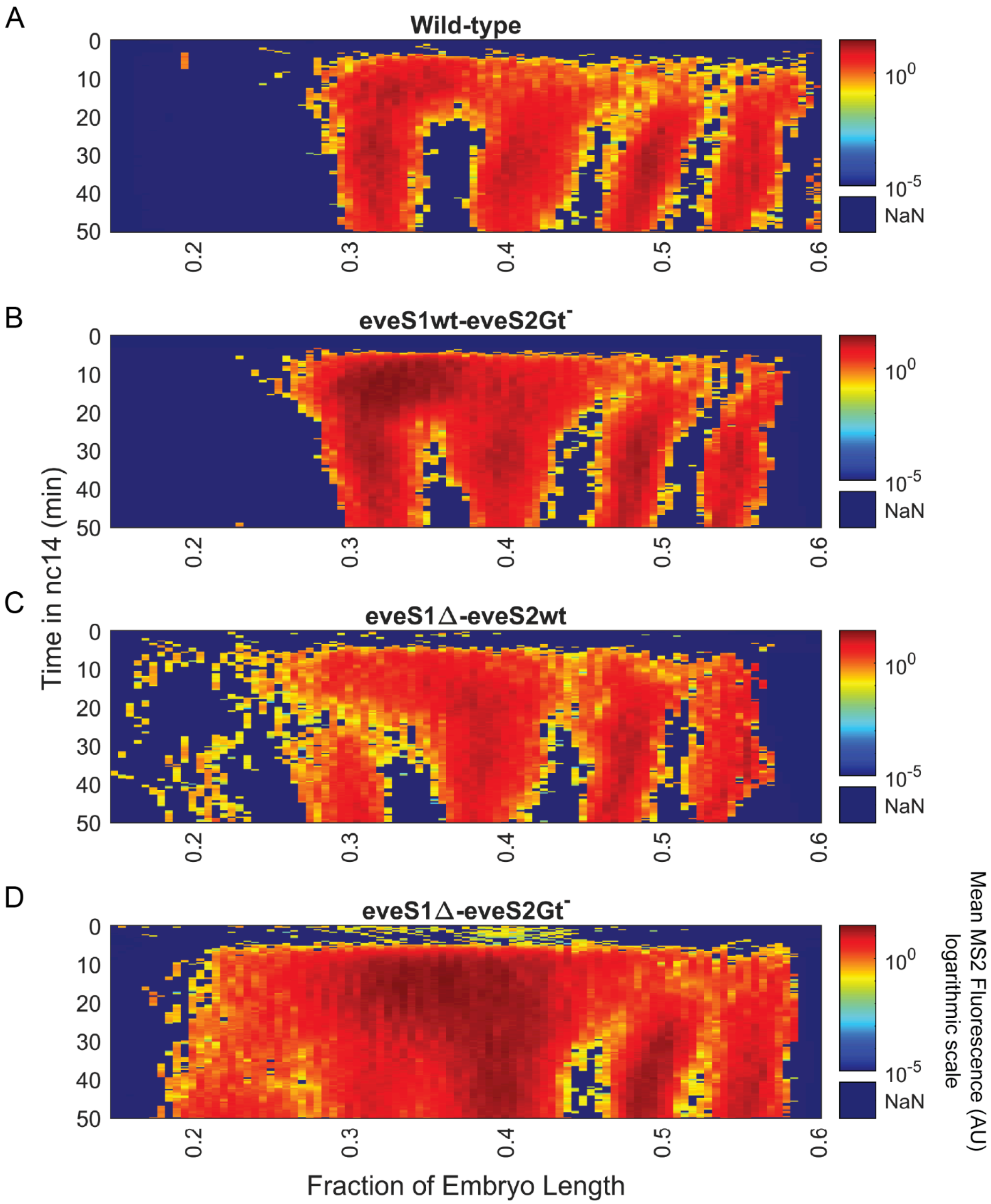

bioRxiv preprint doi: https://doi.org/10.1101/2023.02.09.527927; this version posted June 25, 2024. The copyright holder for this preprint (which was not certified by peer review) is the author/funder, who has granted bioRxiv a license to display the preprint in perpetuity. It is made available under aCC-BY 4.0 International license.

Supplemental Figure 1: Spatiotemporal dynamics of *eve* expression across wild-type and mutant embryos in logarithmic scale. Heatmaps in Figure 2 (B, C, D, and E) rescaled to logarithmic values. **(A)** Kymograph of average *eve*-MS2 fluorescence drawn from 5 eveMS2-BAC (wild-type) embryos.
(B) Average *eve*-MS2 fluorescence from 6 eveS1wt-eveS2Gt^{-} embryos. Inter-stripe transcription between *eve* stripe 1 and *eve* stripe 2 lasts for longer than in wild-type embryos. **(C)** Average *eve*-MS2 fluorescence from 5 eveS1Δ-eveS2wt embryos. Mild expression of *eve* stripe 1 (0.3 fraction of embryo length) and *eve* stripe 0 (0.2 fraction of embryo length) is more apparent on this logarithmic scale. **(D)** Average *eve*-MS2 fluorescence from 6 eveS1Δ-eveS2Gt^{-} embryos. An almost continuous *eve* expression expands from *eve* stripe 2 (0.4 fraction of embryo length) to *eve* stripe 0 (0.2 fraction of embryo length).

bioRxiv preprint doi: https://doi.org/10.1101/2023.02.09.527927; this version posted June 25, 2024. The copyright holder for this preprint (which was not certified by peer review) is the author/funder, who has granted bioRxiv a license to display the preprint in perpetuity. It is made available under aCC-BY 4.0 International license.

Supplemental Figure 2

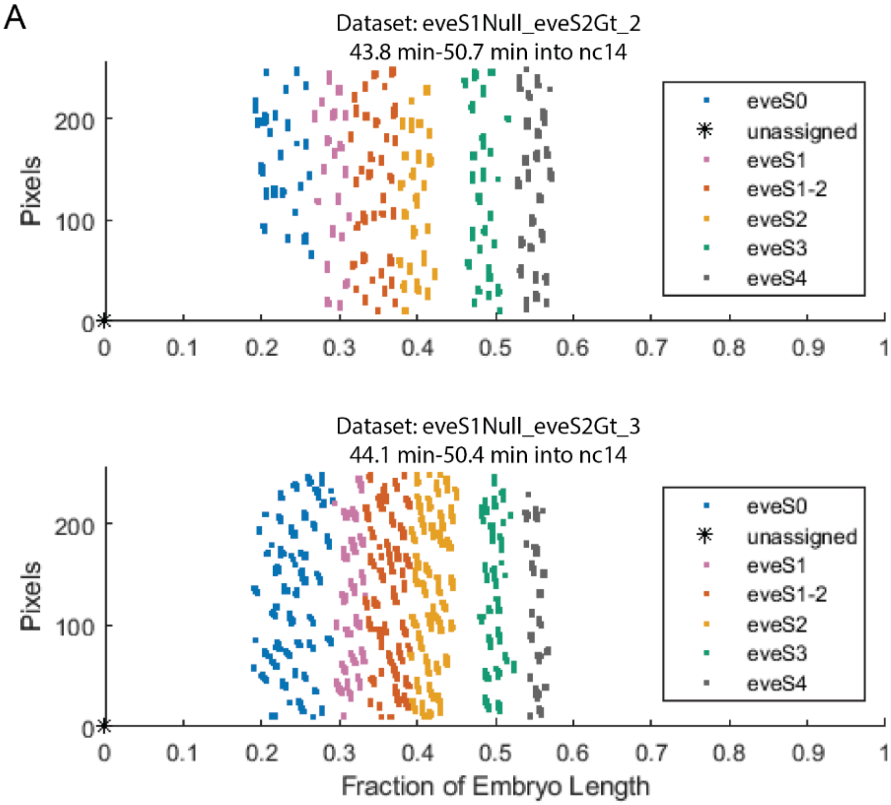

For cpHMM analysis in B and C, we used MS2-bursting data from six eveS1Δ-eveS2Gt⁻ embryos collected during their entire nc14.

In A, we only show snapshots of MS2-foci from two embryos collected between ~44 min and 50 min after the onset of nc14.

B

eve stripe 1-2 bins

50 nuclei
2780 data points
mean fluorescence
4.0513 AU
cpHMM
k_{on} = 0.5143
k_{off} = 0.8487
r = 4.7748

46 nuclei
2818 data points
mean fluorescence
9.6894 AU
cpHMM
k_{on} = 1.0024
k_{off} = 0.7320
r = 8.1682

46 nuclei
2870 data points
mean fluorescence
16.2248 AU
cpHMM
k_{on} = 1.0219
k_{off} = 0.5712
r = 11.5179

eve stripe 2 bins

58 nuclei
2679 data points
mean fluorescence
5.0195 AU
cpHMM
k_{on} = 0.8495
k_{off} = 0.8446
r = 4.9494

48 nuclei
2687 data points
mean fluorescence
9.9743 AU
cpHMM
k_{on} = 1.3070
k_{off} = 0.6535
r = 7.3788

42 nuclei
2693 data points
mean fluorescence
14.6206 AU
cpHMM
k_{on} = 1.6562
k_{off} = 0.5405
r = 9.2305

39 nuclei
2705 data points
mean fluorescence
17.9574 AU
cpHMM
k_{on} = 1.9850
k_{off} = 0.5461
r = 10.9829

39 nuclei
2669 data points
mean fluorescence
21.6730 AU
cpHMM
k_{on} = 1.8322
k_{off} = 0.4239
r = 12.2435

40 nuclei
2728 data points
mean fluorescence
26.1101 AU
cpHMM
k_{on} = 1.9642
k_{off} = 0.3770
r = 14.2191

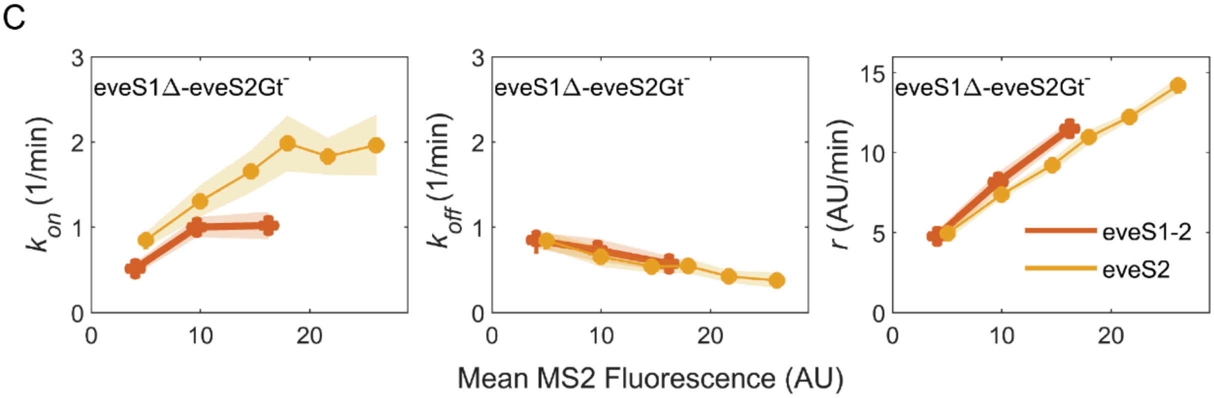

bioRxiv preprint doi: https://doi.org/10.1101/2023.02.09.527927; this version posted June 25, 2024. The copyright holder for this preprint (which was not certified by peer review) is the author/funder, who has granted bioRxiv a license to display the preprint in perpetuity. It is made available under aCC-BY 4.0 International license.

Supplemental Figure 2: Pipeline for the quantification of *eve* bursting parameters (k_{on}, k_{off}, r) in nuclei grouped by stripe and binned by mean MS2 fluorescence (Figure 3). (A) Nuclei in embryos of the same genotype were assigned to a stripe as described in the main text. Here, as an illustrative example, we will follow the analysis of inter stripe 1-2 (vermillion) and *eve* stripe 2 (yellow) in eveS1Δ-eveS2Gt$^-$ embryos. **(B)** Nuclei in *eve* interstripe 1-2 were sorted in three bins of 46-50 nuclei and ~2800 data points according to their mean MS2 fluorescence (4.05, 9.68, and 16.22 AU). Nuclei in *eve* stripe 2 were sorted in six bins of 39-58 nuclei and ~2700 data points according to their mean MS2 fluorescence (5.01, 9.97, 14.62, 17.95, 21.67, and 26.11 AU). Bursting parameters (k_{on}, k_{off}, and r) were calculated for each bin using the cpHMM by (Lammers et al., 2020). This analysis was performed with data from six eveS1Δ-eveS2Gt$^-$ embryos. **(C)** Our analysis makes it possible to plot bursting parameters (y-axis) against mean MS2 fluorescence (x-axis) of each bin.

Complementary Analysis of Bursting Parameters

Bursting parameters in endogenous stripes controlled by mutant enhancers

Some stripes in this work are driven by mutant *eve* enhancers. We found that mutated enhancers modulate transcriptional output of endogenous stripes through the same mechanism as their wild-type counterparts: an increase in k_{on} and r, while k_{off} remains largely constant (Supplemental Figure 3). In eveS1wt-eveS2Gt$^-$ embryos (Supplemental Figure 3C), *eve* stripe 2 is driven by a mutant *eve* stripe 2 enhancer. In eveS1Δ-eveS2wt embryos (Supplemental Figure 3D), *eve* stripe 1 is active in the absence of *eve* stripe 1 enhancer, perhaps due to the activity of the late element. In eveS1Δ-eveS2Gt$^-$ embryos (Supplemental Figure 3E), *eve* stripe 2 is driven by a mutant *eve* stripe 2 enhancer and *eve* stripe 1 is active in the absence of *eve* stripe 1 enhancer. In all cases, our findings support the hypothesis that *eve*-regulatory elements employ a unified strategy to modulate transcriptional output. Bursting parameters of *eve* stripe 1 in embryos with a deleted *eve* stripe 1 enhancer (eveS1Δ-eveS2wt; eveS1Δ-eveS2Gt$^-$) are of particular interest, as this expression is most likely activated by the *eve* late element. If this is the case, the *eve* late element would modulate transcriptional output

bioRxiv preprint doi: https://doi.org/10.1101/2023.02.09.527927; this version posted June 25, 2024. The copyright holder for this preprint (which was not certified by peer review) is the author/funder, who has granted bioRxiv a license to display the preprint in perpetuity. It is made available under aCC-BY 4.0 International license.

through the same mechanism as the other enhancers, further underlining the unity of regulatory strategies across different *eve*-regulatory elements.

Comparison of bursting parameters between sets of nuclei grouped in endogenous and ectopic categories

We computed the bursting parameters of 3-6 bins per stripe (Supplemental Table 2), depending on the amount of data obtained (see SI: Supplemental Figure 2 and Inference of Bursting Parameters in Materials and Methods). To rule out the possibility that the observed k_{on}, k_{off}, and r trends were skewed by the small number of bins, we aimed to redo our analysis with more data points per category (endogenous and ectopic), as a way to contrast bursting parameters between whole endogenous and ectopic regions and examine the bursting parameters trends that result from having 6-13 bins per category (Supplemental Table 3).

We pooled together all nuclei from eveS1Δ-eveS2Gt$^-$ embryos into endogenous (*eve* stripe 1, *eve* stripe 2, *eve* stripe 3, *eve* stripe 4) and ectopic sets (eve stripe 0, *eve* inter-stripe 1-2), and binned them by their mean MS2 fluorescence output to infer and compare their bursting parameters. We did the same analysis in wild-type, eveS1wt-eveS2Gt$^-$, and eveS1Δ-eveS2wt embryos. We contrasted the bursting parameters of ectopic nuclei from eveS1Δ-eveS2Gt$^-$ embryos against sets of endogenous nuclei from eveS1Δ-eveS2Gt$^-$ eveS1wt-eveS2Gt$^-$, eveS1Δ-eveS2wt, and wild-type embryos (Supplemental Figure 4) and observed that all of them follow the same bursting strategy. Ectopic nuclei from eveS1Δ-eveS2Gt$^-$ embryos boost transcriptional output through an increase in average k_{on} (Supplemental Figure 4B) and r (Supplemental Figure 4D), while k_{off} remains largely the same, with only a minor drop

bioRxiv preprint doi: https://doi.org/10.1101/2023.02.09.527927; this version posted June 25, 2024. The copyright holder for this preprint (which was not certified by peer review) is the author/funder, who has granted bioRxiv a license to display the preprint in perpetuity. It is made available under aCC-BY 4.0 International license.

at high mean MS2 fluorescence values (Supplemental Figure 4C). The bursting parameters of endogenous nuclei from all the genotypes in this work follow the same trend.

bioRxiv preprint doi: https://doi.org/10.1101/2023.02.09.527927; this version posted June 25, 2024. The copyright holder for this preprint (which was not certified by peer review) is the author/funder, who has granted bioRxiv a license to display the preprint in perpetuity. It is made available under aCC-BY 4.0 International license.

Supplemental Figure 3

A

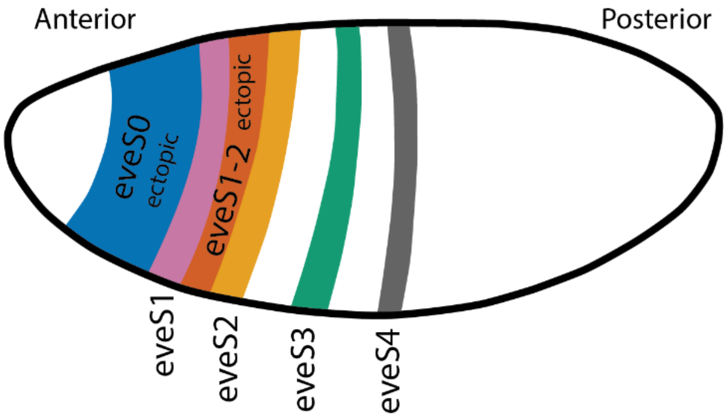

Here, we compare endogenous stripes controlled by wild-type enhancers (solid lines), endogenous stripes controlled by mutant enhancers (dashed lines), and ectopic stripes (dotted lines).

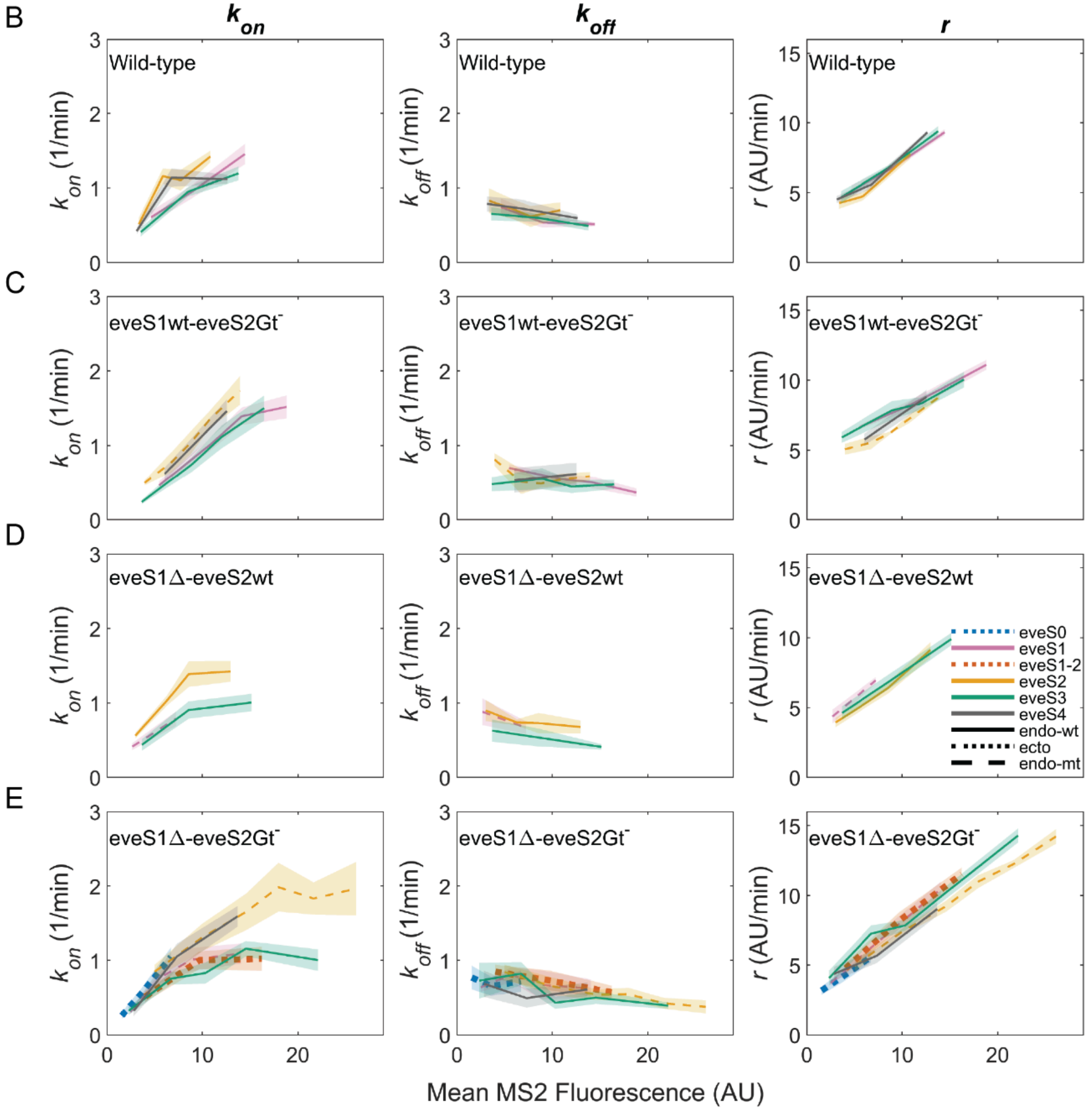

bioRxiv preprint doi: https://doi.org/10.1101/2023.02.09.527927; this version posted June 25, 2024. The copyright holder for this preprint (which was not certified by peer review) is the author/funder, who has granted bioRxiv a license to display the preprint in perpetuity. It is made available under aCC-BY 4.0 International license.

Supplemental Figure 3. Bursting parameter inference for all stripes captured by our data.
We followed the analysis pipeline described in Supplemental Figure 2. k_{on} (*left panels*), k_{off} ,(*middle panels*) and *r* (*right panels*) trends are similar in all endogenous and ectopic stripes in our dataset.
(A) Position and color code of endogenous and ectopic stripes on a fruit fly embryo. **(B)** As previously observed in *eve*-MS2 wild-type embryos (Berrocal et al., 2020), nuclei in all stripes follow the same trends in bursting parameters. All stripes in wild-type embryos are endogenous and are controlled by wild-type enhancers. **(C)** The same trend is observed in all endogenous stripes from eveS1wt-eveS2Gt^{-}, regardless of whether they are controlled by wild-type enhancers (eveS1, eveS3, eveS4); or by mutant enhancers (eveS2). **(D)** endogenous stripes from eveS1Δ-eveS2wt embryos controlled by wild-type (eveS2, eveS3, eveS4) and mutant (eveS1) enhancers display the same trend. **(E)** All endogenous stripes controlled by wild-type (eveS3, eveS4) and mutant (eveS1, eveS2) enhancers, and ectopic stripes (eveS0, eveS1-2) from eveS1Δ-eveS2Gt^{-} embryos share the same bursting strategy.

bioRxiv preprint doi: https://doi.org/10.1101/2023.02.09.527927; this version posted June 25, 2024. The copyright holder for this preprint (which was not certified by peer review) is the author/funder, who has granted bioRxiv a license to display the preprint in perpetuity. It is made available under aCC-BY 4.0 International license.

Supplemental Figure 4

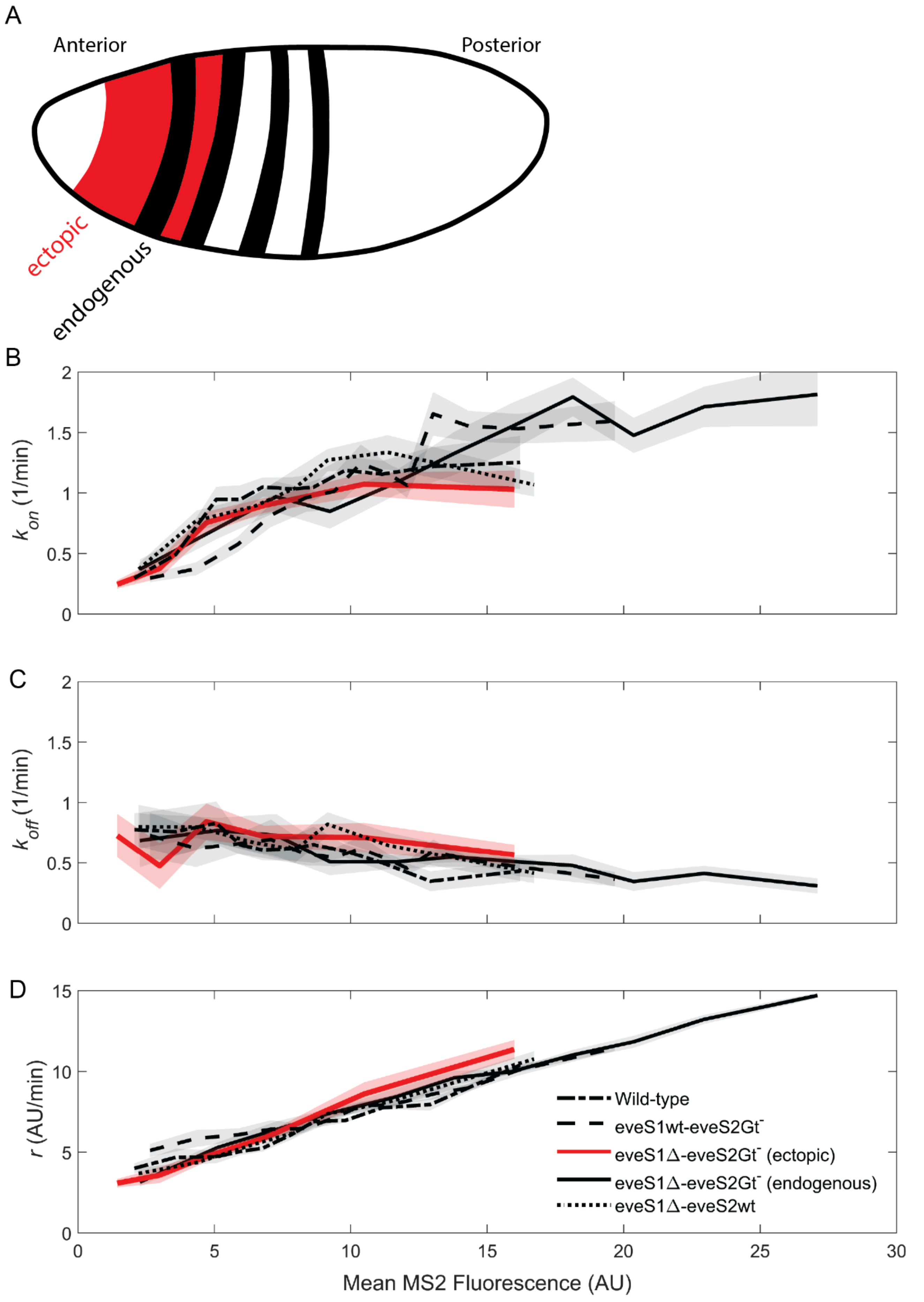

bioRxiv preprint doi: https://doi.org/10.1101/2023.02.09.527927; this version posted June 25, 2024. The copyright holder for this preprint (which was not certified by peer review) is the author/funder, who has granted bioRxiv a license to display the preprint in perpetuity. It is made available under aCC-BY 4.0 International license.

Supplemental Figure 4: Comparison of bursting parameters between endogenous and ectopic gene expression regions. We followed the cpHMM-based analysis pipeline (as described in Supplemental Figure 2) on nuclei grouped in two categories: endogenous (eveS1, eveS2, eveS3, and eveS4) and ectopic (eveS0 and eveS1-2); instead of grouping nuclei by stripes. k_{on}, k_{off}, and r parameters of ectopic (red) and endogenous (black) regions, estimated from nuclei binned by their mean MS2 fluorescence. Ectopic regions (red solid line) from eveS1Δ-eveS2Gt^{-} embryos follow the same bursting strategies as the endogenous regions from all other genotypes: wild-type (black dash-dot line), eveS1Δ-eveS2Gt^{-} (black solid line), eveS1wt-eveS2Gt^{-} (black dashed line), eveS1Δ-eveS2wt (black dotted line). **(A)** Regions where data points were analyzed together under the category endogenous (black) or ectopic (red). **(B)** Average k_{on} values increase in brighter *eve*-active nuclei. **(C)** Average k_{off} values remain constant and have a slight decrease in highly *eve*-active nuclei. **(D)** Average r values increase in brighter *eve*-active nuclei.

Materials and Methods

DNA constructs and fly lines

We generated 4 reporter constructs based on a previously established Bacterial Artificial Chromosome (BAC) carrying the ~20 Kb DNA sequence around *eve (Venken et al., 2006, 2009)*, and whose *eve* coding sequence has been replaced by an MS2::*yellow* transcriptional unit (Berrocal et al., 2020). We used wild-type eveMS2-BAC from (Berrocal et al., 2020). The other 3 BAC constructs were derived from wild-type eveMS2-BAC. These constructs carried mutant versions of *eve* stripe 1 and *eve* stripe 2 enhancers. Vector Builder (https://en.vectorbuilder.com/) generated the mutant versions through ccdB-amp cassette mediated recombineering. These mutant BACs are available on Vector Builder's website. SnapGene (.dna) files with eveMS2 BAC sequences are in the repository https://github.com/aberrocal/BurstingStrategies-eve.git, folder BurstingStrategies-eve/_DataSubmission/BACSequences/.

eveS1wt-eveS2Gt^{-} BAC construct (Vector Builder-Service Proposal: P180328-1009dgs) contains a wild-type *eve* stripe 1 and a mutant version of *eve* stripe 2 enhancer with three Giant-binding sites deleted, as shown in Table I of (Small et al., 1992). We chose

bioRxiv preprint doi: https://doi.org/10.1101/2023.02.09.527927; this version posted June 25, 2024. The copyright holder for this preprint (which was not certified by peer review) is the author/funder, who has granted bioRxiv a license to display the preprint in perpetuity. It is made available under aCC-BY 4.0 International license.

to disrupt the three Gt-binding sites within the *eve* stripe 2 enhancer (Figure 2B) that had previously been tied to ectopic anterior expansion of *eve* stripe 2 expression when deleted in the context of the Minimal Regulatory Element of the eveS2 enhancer (eveS2-MRE) (Small et al., 1992). eveS2-MRE is a 480bp regulatory sequence within the *eve* stripe 2 enhancer (~2kb total length) sufficient to drive the expression of *eve* stripe 2.

eveS1Δ-eveS2Gt$^-$ BAC construct (Vector Builder-Service Proposal: P180614-1002pzr) has the *eve* stripe 1 enhancer, as defined by ChIP-seq data of the enhancer-associated protein Zelda (Harrison et al., 2011), replaced by a ccdB-amp cassette and *eve* stripe 2 enhancer replaced by a mutant version with three Giant binding sites deleted as described above.

eveS1Δ-eveS2wt BAC construct (Vector Builder-Service Proposal: P190605-1001zkt) has *eve* stripe 1 enhancer replaced with a ccdB-amp cassette and a wild-type *eve* stripe 2. To sum up, we used the fly line carrying wild-type eveMS2-BAC from (Berrocal et al., 2020) and we generated 3 new fly lines carrying genome integrations of the aforementioned constructs. The mutant versions of eveMS2-BAC used in this work were inserted in the genome via ϕC31 integrase mediated recombination. Mutant constructs were either sent to BestGene Inc (eveS1wt-eveS2Gt$^-$, eveS1Δ-eveS2wt) for germline injection or injected in our laboratory (eveS1Δ-eveS2Gt$^-$). All constructs integrated into a ϕC31 AttP insertion site in chromosome 3L (Bloomington stock #24871; landing site VK00033; cytological location 65B2).

bioRxiv preprint doi: https://doi.org/10.1101/2023.02.09.527927; this version posted June 25, 2024. The copyright holder for this preprint (which was not certified by peer review) is the author/funder, who has granted bioRxiv a license to display the preprint in perpetuity. It is made available under aCC-BY 4.0 International license.

Imaging

We crossed male flies from lines carrying eveMS2-BAC constructs (w-; +; MS2::yellow) and female flies carrying His::RFP and MCP::GFP fusion proteins (yw; His::RFP; MCP::GFP) (Garcia et al., 2013). His::RFP allows for visualization of nuclei, MCP::GFP binds MS2 nascent transcripts to form fluorescent puncta at sites of nascent MS2 transcription. We set embryo-collection cages with ~30 male and ~100 female fruit flies, and collected offspring embryos after 1h 30min. All movies in the same dataset were recorded within ~1 week. We mounted embryos on a slide for confocal imaging, as described in (Berrocal et al., 2020; Bothma et al., 2014). Aging embryos for 1h 30min allows us to capture the entire interval between the 14th synchronous cell cleavage and the beginning of gastrulation. We recorded a total of 22 live embryos as shown in Supplemental Table 1. All imaging was done in a Zeiss-800 scanning-laser confocal microscope. Movies of embryonic development were captured under a 63x oil objective, in windows of 202.8 μm x 50.7 μm, at pixel size of 0.2 μm, zoom 0.5x. Movies were recorded in two channels, EGFP for MS2 signal, and TagRFP for His::RFP signal. Imaging parameters were 16 bits per pixel, scan mode frame, bidirectional scanning, scan speed 7, pixel dwelling 1.03 μsec, laser scanning averaging 2, averaging method mean, averaging mode line, laser power EGFP 30 μW and TagRFP 7.5 μW, master gain in EGFP channel 550V and in TagRFP channel 650V, digital offset in both channels 0, digital gain in both channels 1, pinhole size 44 μm (1 Airy unit - 0.7 μm/section) at 63x objective, laser filters EGFP:SP545 and TagRFP:LBF640. Data points consist of Z-stacks of 21 slices separated by intervals of 0.5 μm, to span a range of 10 μm across the Z axis. Z-stack mode full stack. Whole Z-stacks were recorded every 16.8 sec

bioRxiv preprint doi: https://doi.org/10.1101/2023.02.09.527927; this version posted June 25, 2024. The copyright holder for this preprint (which was not certified by peer review) is the author/funder, who has granted bioRxiv a license to display the preprint in perpetuity. It is made available under aCC-BY 4.0 International license.

(wild-type, eveS1wt-eveS2Gt$^-$, eveS1Δ-eveS2Gt$^-$) and 19.5 sec (eveS1Δ-eveS2wt). The difference in time resolution between datasets does not impact our analysis, as the cpHMM analyzes interpolated data points at 20 s intervals. These parameters are based on the imaging protocol and settings in (Berrocal et al., 2020). We stopped live imaging of individual embryos after 50 min into nuclear cycle 14, before the cell rearrangements of gastrulation, and took mid-sagittal and surface images of the whole embryo to localize our 202.8 μm x 50.7 μm window along the embryonic anterior-posterior axis. Raw data from confocal microscope imaging is publicly available in Zenodo (https://zenodo.org/, https://doi.org/10.5281/zenodo.7204096) (see SI section: Data and Code).

bioRxiv preprint doi: https://doi.org/10.1101/2023.02.09.527927; this version posted June 25, 2024. The copyright holder for this preprint (which was not certified by peer review) is the author/funder, who has granted bioRxiv a license to display the preprint in perpetuity. It is made available under aCC-BY 4.0 International license.

Supplemental Table 1

**Wild-type datasets**	**Stripes Recorded**
eveS1wt_eveS2wt_1	eveS1, eveS2, eveS3, eveS4
eveS1wt_eveS2wt_2	eveS1, eveS2, eveS3, (eveS4)
eveS1wt_eveS2wt_3*	eveS1, eveS2, eveS3, eveS4
eveS1wt_eveS2wt_4	eveS1, eveS2, eveS3, eveS4
eveS1wt_eveS2wt_5	eveS1, eveS2, eveS3, eveS4, (eveS5)
**eveS1wt-eveS2Gt$^-$ datasets**	**Stripes Recorded**
eveS1wt_eveS2Gt_1	eveS1, eveS1-2, eveS2, eveS3
eveS1wt_eveS2Gt_2	eveS1, eveS1-2, eveS2, eveS3, (eveS4)
eveS1wt_eveS2Gt_3	eveS1, eveS1-2, eveS2, eveS3, eveS4
eveS1wt_eveS2Gt_4	eveS1, eveS1-2, eveS2, eveS3
eveS1wt_eveS2Gt_5*	eveS1, eveS1-2, eveS2, eveS3, eveS4
eveS1wt_eveS2Gt_6	eveS1, eveS1-2, eveS2, eveS3, eveS4
**eveS1Δ-eveS2wt datasets**	**Stripes Recorded**
eveS1Null_eveS2wt_1	(eveS0), eveS1, eveS2, eveS3, eveS4
eveS1Null_eveS2wt_2*	eveS0, eveS1, eveS2, eveS3
eveS1Null_eveS2wt_3	eveS0, eveS1, eveS2, eveS3, (eveS4)
eveS1Null_eveS2wt_4	(eveS0), eveS1, eveS2, eveS3, (eveS4)
eveS1Null_eveS2wt_5	eveS0, eveS1, eveS2, eveS3
**eveS1Δ-eveS2Gt$^-$ datasets**	**Stripes Recorded**
eveS1Null_eveS2Gt_1	eveS0, eveS1, eveS1-2, eveS2, eveS3, eveS4
eveS1Null_eveS2Gt_2	eveS0, eveS1, eveS1-2, eveS2, eveS3, eveS4
eveS1Null_eveS2Gt_3	eveS0, eveS1, eveS1-2, eveS2, eveS3, eveS4
eveS1Null_eveS2Gt_4	eveS0, eveS1, eveS1-2, eveS2, eveS3, eveS4
eveS1Null_eveS2Gt_5*	eveS0, eveS1, eveS1-2, eveS2, eveS3
eveS1Null_eveS2Gt_6	eveS0, eveS1, eveS1-2, eveS2, eveS3

bioRxiv preprint doi: https://doi.org/10.1101/2023.02.09.527927; this version posted June 25, 2024. The copyright holder for this preprint (which was not certified by peer review) is the author/funder, who has granted bioRxiv a license to display the preprint in perpetuity. It is made available under aCC-BY 4.0 International license.

Supplemental Table 1: Datasets and stripes. We recorded 5 wild-type eveMS2-BAC (eveS1wt-eveS2wt) datasets, 6 eveS1wt-eveS2Gt^{-} (eveS1wt_eveS2Gt), 5 eveS1Δ-eveS2wt (eveS1Null_eveS2wt), and 6 eveS1Δ-eveS2Gt^{-} (eveS1Null_eveS2Gt) for a total of 22 datasets. Movies in every dataset capture between 3 and 6 stripes. Supplemental Table 1 shows stripes captured in each dataset. Stripes in parentheses had few active nuclei (eveS0) or were not captured in their entirety (eveS4) and (eveS5). Asterisks indicate datasets used for stills in Figure 2.

Segmentation and quantification of movies

We tracked MS2 foci from movies and segmented them using the MATLAB based analysis pipeline developed by (Berrocal et al., 2020; Garcia et al., 2013; Lammers et al., 2020). Specifically, for segmentation of MS2/MCP::GFP foci across stacks on the Z-axis, we combined the MATLAB pipeline mentioned above with Fiji-Weka Segmentation 3D software, as described in (Berrocal et al., 2020). The MATLAB/Fiji-Weka pipeline extracts the position of nuclei and the fluorescence intensity and position of individual MS2 foci over time. The final result of the MATLAB based analysis pipeline are CompiledParticles.mat files that contain the position of nuclei, as well as their MS2 fluorescence intensity over time (see Data and Code).

Assignment of *eve*-active nuclei to stripes

We manually segmented nuclei from eveS1Δ-eveS2Gt^{-} and eveS1wt-eveS2Gt^{-} fly lines, as their stripes were not always clearly discernible. For these embryos, we assigned nuclei to individual stripes based on the position of stripes at 45 min into nc14, when they became separated from the background. The boundary between *eve* stripe 1-2 and *eve* stripe 2 in eveS1Δ-eveS2Gt^{-} embryos was set at 36% of embryo length, according to the kymograph of MS2 fluorescence over time. On the other hand, eveS1Δ-eveS2wt and wild-type embryos showed defined stripes after 25 min into nc14. Thus, we used a MATLAB k-means clustering algorithm to dynamically assign *eve*-active nuclei to

bioRxiv preprint doi: https://doi.org/10.1101/2023.02.09.527927; this version posted June 25, 2024. The copyright holder for this preprint (which was not certified by peer review) is the author/funder, who has granted bioRxiv a license to display the preprint in perpetuity. It is made available under aCC-BY 4.0 International license.

individual stripes, tracking nuclei by the accumulation of MS2 fluorescent output in windows of five-minutes. Nuclei active between 0 and 25 min into nc14 were assigned to stripes based on their position at 25 min into nc14. We generated movies of segmented MS2 spots assigned to individual stripes in windows of ~5 minutes. MATLAB scripts for manual and k-means-automated segmentation of stripes, as well as scripts to generate movies of segmented stripes are available in github (see Data and Code).

Generation of heatmaps in Figure 2 and Supplemental Figure 1

We used traces of MS2 fluorescence intensity over time, which reflect transcriptional activity, to generate heatmap/kymographs of MS2 transcription datasets. We generated heatmaps (Figure 2, Supplemental Figure 1) by collapsing data points from all embryos of the same genotype into a single kymograph plot. We started by adjusting the position of nuclei in each embryo relative to nuclei in other embryos of the same genotype. As we had assigned MS2 active nuclei to individual stripes, we measured the distance along the anterior-posterior axis from each MS2 focus to the center of its corresponding stripe. We inferred the position of pseudo-stripes formed by the combined data from all embryos of the same genotype. We calculated the position of pseudo-stripes along the anterior-posterior embryo axis by averaging the position of the center of stripes along the anterior-posterior axis in individual embryos of the same genotype. Finally, we assigned a position to all nuclei of the same genotype relative to pseudo-stripes by positioning them at the same distance from the center of pseudo-stripes as they were from the center of the stripe where they originated. We followed the same procedure to locate the position of inactive nuclei.

bioRxiv preprint doi: https://doi.org/10.1101/2023.02.09.527927; this version posted June 25, 2024. The copyright holder for this preprint (which was not certified by peer review) is the author/funder, who has granted bioRxiv a license to display the preprint in perpetuity. It is made available under aCC-BY 4.0 International license.

Labeling *eve* patterns as endogenous or ectopic

To compare the bursting parameters between endogenous and ectopic regions of *eve* activity, we segmented MS2-active nuclei and assigned them to individual regions that were deemed to be either endogenous or ectopic. We labeled regions as endogenous if their position overlapped within the boundaries of wild-type *eve* stripes (*eve* stripe 1, *eve* stripe 2, *eve* stripe 3, *eve* stripe 4); or as ectopic if their position overlapped with the inter-stripe region between *eve* stripe 1 and *eve* stripe 2 (*eve* stripe 1-2) or with the novel *eve* stripe 0 (~20% embryo length). All stripes in wild-type embryos were labeled as endogenous.

Selection of a three-state model of promoter activity and a compound Hidden Markov Model for inference of promoter states from MS2 fluorescent signal

We selected a three-state model of promoter activity (OFF, ON_1, ON_2) based on the following argument. Transcription in pre-gastrulating *Drosophila* embryos occurs after DNA replication, and sister chromatids remain paired. However, most of the time, paired MS2-tagged sister loci cannot be resolved independently using diffraction-limited microscopy (Lammers et al., 2020). Therefore, each fluorescent spot in our data results from the combined activity of two promoters, each of which, in the simplest possible model of transcriptional bursting, may be ON or OFF (Lammers et al., 2020). To account for this, the cpHMM infers three states from the observed MS2 data: OFF (both sister promoters inactive), ON_1 (one sister promoter active), and ON_2 (two sister promoters active). For ease of presentation, we aggregated ON_1 and ON_2 states into a single effective ON state, as we did in our previous work (Berrocal et al., 2020). This leads to

bioRxiv preprint doi: https://doi.org/10.1101/2023.02.09.527927; this version posted June 25, 2024. The copyright holder for this preprint (which was not certified by peer review) is the author/funder, who has granted bioRxiv a license to display the preprint in perpetuity. It is made available under aCC-BY 4.0 International license.

an effective two-state model with one OFF and one ON state and three burst parameters: k_{off}^{-1} (the burst duration), k_{on} (the burst frequency), and r (the burst amplitude). k_{on} is defined as the sum of the transition rates from OFF to any of the two active states described above: OFF → ON_1 and OFF → ON_2. k_{off} is defined as the rate at which the system returns to the OFF state upon leaving it, which is described by the formula $k_{off}^{-1} = (\frac{1}{p_{off}} - 1)\, k_{on}^{-1}$, where p_{off} is the fraction of time the system spends in the OFF state. k_{off} is the inverse of mean burst duration. r is defined by the average of the rates of transcription initiation in the two ON states (r_1 and r_2) weighted by the fraction of the time that the system spends on each state (p_1 and p_2) as described by the formula $r = \frac{p_1 r_1 + p_2 r_2}{p_1 + p_2}$ (Lammers et al., 2020). The outputs of the three state model of promoter activity (k_{on}, k_{off}, and r) were used for downstream analyses.

The three-state model of promoter activity is the simplest model compatible with our current understanding of transcription at the *eve* locus in early fruit fly embryos. However, we do not dismiss the possibility that more complex processes, not captured by our model, define *eve* transcription. Promoters, for instance, may exhibit more than two states of activity, beyond a simple ON and OFF mechanism. Nevertheless, as pointed out by (Lammers et al., 2020) - SI Section: G. cpHMM inference sensitivities) cross-validation of different model schemes (two, three, or multiple state Hidden Markov Models) do not yield consistent results regarding on which one is more accurate; and for the time being, there is no alternative to a HMM for inference of promoter states from MS2/PP7 fluorescence signals obtained using laser-scanning confocal microscopy (Lammers et al., 2020; Syed et al., 2023) (although other approaches exist using

bioRxiv preprint doi: https://doi.org/10.1101/2023.02.09.527927; this version posted June 25, 2024. The copyright holder for this preprint (which was not certified by peer review) is the author/funder, who has granted bioRxiv a license to display the preprint in perpetuity. It is made available under aCC-BY 4.0 International license.

state-of-the-art microscopy and deconvolution algorithms to improve signal-to-noise ratio). Furthermore, orthogonal approaches to quantify transcription that rely on static methods, such as smFISH, have a limited ability to capture temporal dynamics. Due to these considerations, we selected a HMM based on an effective two-state model (derived from a three-state model) of promoter activity to describe our live MS2 imaging data.

Inference of bursting parameters

We used a cpHMM approach (Lammers et al., 2020) to extract average bursting parameters (k_{on}, k_{off}, r) from different sets of MS2-active nuclei. We input MS2 fluorescent traces over time from these sets into the cpHMM. Specifically, we combined nuclei from same-genotype embryos, sorted them by stripe and distributed them across bins of varying fluorescence. To ensure reliable inference, we enforced each bin to contain ~40 nuclei, equivalent to ~2500 time points at a 20 sec resolution (Lammers et al., 2020). The number of bins was determined by the amount of data available (Supplemental Table 2).

Wild-type embryos yielded sufficient nuclei to support the cpHMM inference of bursting parameters for various endogenous stripes (*eve* stripe 1, 2, 3, 4). eveS1wt-eveS2Gt$^-$ and eveS1Δ-eveS2wt did not yield enough ectopically active nuclei for cpHMM analysis (*eve* stripe 1-2 in eveS1wt-eveS2Gt$^-$; *eve* stripe 0 in eveS1Δ-eveS2wt). These fly lines did exhibit endogenous *eve* stripes with enough active-nuclei for further analysis on the cpHMM (*eve* stripe 1, 2, 3, and 4 in eveS1wt-eveS2Gt$^-$; *eve* stripe 1, 2, and 3 in

bioRxiv preprint doi: https://doi.org/10.1101/2023.02.09.527927; this version posted June 25, 2024. The copyright holder for this preprint (which was not certified by peer review) is the author/funder, who has granted bioRxiv a license to display the preprint in perpetuity. It is made available under aCC-BY 4.0 International license.

eveS1Δ-eveS2wt). eveS1Δ-eveS2Gt$^-$ embryos did yield sufficient *eve*-active nuclei (297 nuclei) to support cpHMM inference of the bursting parameters of ectopic *eve* stripe 1-2 and *eve* stripe 0. It also resulted in enough active nuclei for the cpHMM inference of bursting parameters of endogenous stripes (*eve* stripe 1, 2, 3, and 4).

The output of the effective two-state cpHMM described above are the bursting parameters (k_{on}, k_{off}, r) for each set of nuclei input into the model. Thus, Figure 3 and Supplemental Figure 3 are plots of mean k_{on}, k_{off}, r, and their standard deviations σ_{kon}, σ_{koff}, σ_r, computed from sets of nuclei binned by stripe. For Supplemental Figure 4, we followed a similar approach, but grouping active nuclei by their endogenous or ectopic location. Nuclei grouped in endogenous and ectopic categories were distributed across 6-13 bins of increasing fluorescence (Supplemental Table 3). Their mean k_{on}, k_{off}, r, and standard deviations, σ_{kon}, σ_{koff}, σ_r were plotted in Supplemental Figure 4.

bioRxiv preprint doi: https://doi.org/10.1101/2023.02.09.527927; this version posted June 25, 2024. The copyright holder for this preprint (which was not certified by peer review) is the author/funder, who has granted bioRxiv a license to display the preprint in perpetuity. It is made available under aCC-BY 4.0 International license.

Supplemental Table 2

**Wild-type - Stripes**	**Number of bins**
eveS1	3
eveS2	4
eveS3	3
eveS4	3
eveS5	0
**eveS1wt-eveS2Gt$^-$ - Stripes**	**Number of bins**
eveS1	4
eveS1-2	0
eveS2	5
eveS3	4
eveS4	2
**eveS1Δ-eveS2wt - Stripes**	**Number of bins**
eveS0	0
eveS1	2
eveS2	4
eveS3	3
eveS4	1
**eveS1Δ-eveS2Gt$^-$ - Stripes**	**Number of bins**
eveS0	3
eveS1	4
eveS1-2	3
eveS2	6
eveS3	5
eveS4	3

bioRxiv preprint doi: https://doi.org/10.1101/2023.02.09.527927; this version posted June 25, 2024. The copyright holder for this preprint (which was not certified by peer review) is the author/funder, who has granted bioRxiv a license to display the preprint in perpetuity. It is made available under aCC-BY 4.0 International license.

Supplemental Table 2: Binning by stripe. We pooled together nuclei from all embryos per dataset, sorted them by the stripe where they are located and distributed them in bins of varying fluorescence. Each bin contains ~40 nuclei (~2500 time points). E.g., all nuclei in *eve* stripe 1 (eveS1) from the five *eve* wild-type embryos in our dataset were assigned to 3 bins according to their mean MS2 fluorescence, as each bin must contain ~40 nuclei, or ~2500 data points, for input into the cpHMM.

Supplemental Table 3

**Wild-type**	**Number of Bins**
Ectopic	0
Endogenous	11
**eveS1wt-eveS2Gt$^-$**	**Number of Bins**
Ectopic	0
Endogenous	13
**eveS1Δ-eveS2wt**	**Number of Bins**
Ectopic	0
Endogenous	7
**eveS1Δ-eveS2Gt$^-$**	**Number of Bins**
Ectopic	6
Endogenous	11

Supplemental Table 3: Binning by endogenous/ectopic. We pooled together nuclei from all embryos per dataset, sorted them by endogenous or ectopic, according to whether the stripe where they were located was deemed endogenous or ectopic, and distributed them in bins of varying fluorescence. Each bin contains ~40 nuclei (~2500 time points). E.g. All endogenous nuclei in the 5 *eve* wild-type embryos were distributed among 11 bins of increasing MS2 fluorescence. Some datasets have their ectopic bin empty, as they had less than ~40 active nuclei in their ectopic regions.
